# A Multiscale Constraints Method Localization of 3D Facial Feature Points

**DOI:** 10.1155/2015/178102

**Published:** 2015-10-11

**Authors:** Hong-an Li, Yongxin Zhang, Zhanli Li, Huilin Li

**Affiliations:** ^1^College of Computer Science and Technology, Xi'an University of Science and Technology, Xi'an 710054, China; ^2^School of Mechanical Engineering, Xi'an University of Science and Technology, Xi'an 710054, China; ^3^School of Information Technology, Luoyang Normal University, Luoyang 471022, China

## Abstract

It is an important task to locate facial feature points due to the widespread application of 3D human face models in medical fields. In this paper, we propose a 3D facial feature point localization method that combines the relative angle histograms with multiscale constraints. Firstly, the relative angle histogram of each vertex in a 3D point distribution model is calculated; then the cluster set of the facial feature points is determined using the cluster algorithm. Finally, the feature points are located precisely according to multiscale integral features. The experimental results show that the feature point localization accuracy of this algorithm is better than that of the localization method using the relative angle histograms.

## 1. Introduction

With the development of 3D information acquisition technology, the research of 3D facial feature has gained more and more extensive attention. The automatic localization of the facial feature points is a study hotspot in the field of medical computer vision, which is a precondition for face recognition, face animation, face tracking, and 3D face reconstruction. At present, the 3D facial feature point localization algorithms have not been researched in depth though the 2D facial feature point localization algorithms have matured. Wang et al. [1] applied the Jet Bunch algorithm of 2D facial feature point localization to the process of 3D facial feature point localization; Xu et al. [2] proposed a hierarchical filtering algorithm, which could locate the feature points of nasal tip. While these methods are beneficial to detect other facial key feature points, they are insensitive to noises and have the rotation and translation invariance characters with a poor generality, and the localization results are unsatisfactory except the nasal tip feature points. Feng et al. [3] proposed a feature point localization algorithm based on relative angle histograms. In a 3D point distribution model, the relative angle histograms of all points are calculated firstly, and then the features are located according to the similarity of the histograms. The algorithm is of high efficiency and stability, but it locates the feature points just in a small scope with an inaccurate result. Li and Da [4] suggested a 3D facial feature point localization algorithm that combined a priori knowledge with differential characteristics, but the result is not satisfactory because of the unique and complex diversity of the face model.

In order to solve these problems, we put forward a 3D facial feature point localization method based on the relative angle histograms and multiscale constraints. The cluster point set of the facial features is created firstly, and then the multiscale integral characteristics are used to locate the feature points accurately. As a result, the accuracy of the feature point localization is improved.

## 2. Model Preprocessing

### 2.1. Coordinate System Transformation

CT (computed tomography) images are acquired from living samples in a hospital, whose contour lines are extracted using the method of combining the improved snake algorithm with the ray method [5]. In order to obtain the 3D face sample that is constructed by a single layer triangular mesh, the 3D surface is reconstructed using the Ganapathy algorithm to connect the neighbor contour lines [6]. When the CT data are collected in a hospital, the postures of every body's head are different, and unavoidably the reconstructed single layer 3D models would lie in different coordinate systems. For the convenience of defining the feature points, all the models are unified to a uniform coordinate system.

A face model (FM) is usually constructed by a triangular mesh which is composed of vertexes (*V*) and triangular facets (*F*), so FM could be expressed as a linear representation, FM = (*V*, *F*). *v*_*i*_ is the *i*th point of the FM, 1 ≤ *i* ≤ *N*, and the coordinate of *v*_*i*_ is expressed by *v*_*i*_ = (*x*_*i*_, *y*_*i*_, *z*_*i*_)^*T*^. The center of the FM is computed by *O* = (1/*N*)∑_*i*=1_^*N*^ *v*_*i*_, and then the point set *V* is transformed into the coordinate system whose origin is *O*.

The point set *V* is taken as a 3D random variable, and a positive definite covariance matrix *C* could be obtained from the transformed *V*. Three biggest eigenvalues *λ*_1_, *λ*_2_, *λ*_3_  (*λ*_1_ ≥ *λ*_2_ ≥ *λ*_3_ ≥ 0) of *C* are calculated, and the eigenvector responding to the eigenvalues is *E* = [*e*_1_, *e*_2_, *e*_3_], where *E* is an orthogonal matrix, and *E* ^*T*^ *E* = *I*, where *I* is a unit matrix.

According to the related knowledge of algebra, *CE* = *λE*, *C* could be calculated out. Define *U* as a linear combination of *V*, *U* = *E* ^*T*^(*V* − *O*), and *U* is orthogonalised through the equation Cov (*U*) = *E* ^*T*^ *CE* = Λ, and then the variance among sets *U*_*i*_ could be expressed as

(1) $$\begin{array}{c} Cov\;\left(\;U_{i},U_{j}\;\right)=Cov\;\left(\;e_{i}^{T},e_{j}^{T}\;\right)=e_{i}^{T}Ce_{j}^{i}=\left\{\begin{array}{cc} \lambda_{i} & i=j\\ 0 & i\neq j. \end{array}\right. \end{array}$$

**Figure 1:**
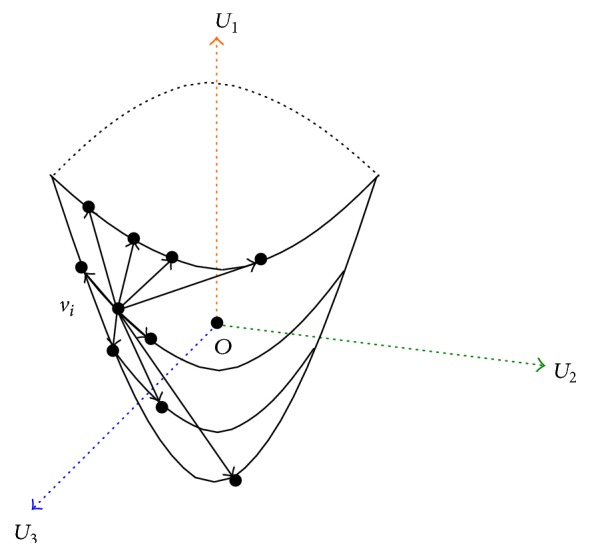
The coordinate system of a model.

*U*_*i*_  (1 ≤ *i* ≤ 3) is taken as the *i*th principal component of variable *V*, and then we construct the coordinate system shown in Figure 1, whose origin is *O* and coordinate axes are *U*_1_, *U*_2_, *U*_3_.

### 2.2. Feature Point Definition

**Figure 2:**
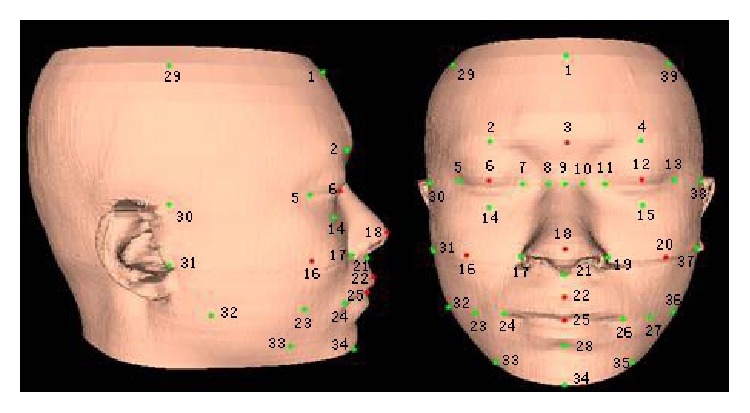
Feature points of the single layer face model.

The feature points of a 3D face must have connotations and lie in a cognizable key position. In the same sample the feature points are distinct from their neighbor points on geometrical characteristics, while in different samples the features of the same feature points are similar. To meet the experimental requirements, a total of 39 feature points covering the front half face are defined according to the knowledge of anthropology, anatomy, and the feature points defined by MPEG4 expert group [7, 8]. The positions of the feature points are shown in Figure 2.

## 3. Feature Point Cluster Based on the Relative Angle Histograms

### 3.1. Relative Angle Histograms

After the face model is unified into a uniform coordinate system, the next step is to calculate the relative angle histograms of points in the face model. According to Feng et al. [3], any point *v*_*i*_ of the face model expresses the angle between vector $\vec{v_{i}v_{j}}\;\;(1\leq j\leq N,\;\;j\neq N)$ and *U*_1_, and after the angle is transformed its value range is [0,2*π*].

The relative angles of the point in the face model describe a spatial relationship between every point, which possesses rotation, translation, and scaling invariance and better noise robustness. Any point of the model has *N* − 1 relative angles and then has *N* − 1 relative angle distributions. The relative distributions have statistical characteristics, and the distribution curves describe in detail the global characteristics of every point in the model.

**Figure 3:**
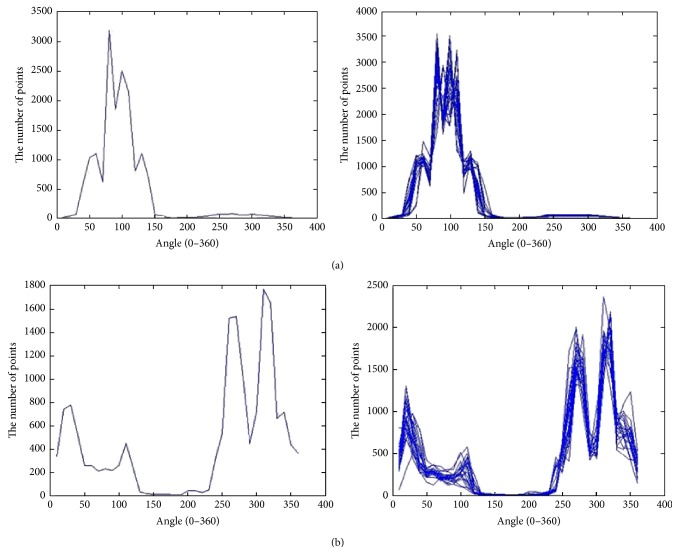
The relative angle distribution of the feature points. (a) The feature points of the forehead center: the left is the relative angle distribution of forehead center feature points and the right is the relative angle distribution of superimposed forehead center feature points. (b) The feature points of the right mouth corner: the left is the relative angle distribution of right mouth corner feature points and the right is the relative angle distribution of superimposed right mouth corner feature points.

Calculate the *j*th relative angle Ang_*i*,*j*_  (1 ≤ *j* ≤ *N*, *i* ≠ *j*) of any point *v*_*i*_ in the model. The value range [0,2*π*] is divided into 40 parts. Count the number of points in every region for every Ang_*i*,*j*_, and the statistical result is the relative angle histogram of point *v*_*i*_.  Figure 3 shows the feature points' relative angle distributions of the forehead center and right mouth corner as well as those which are superimposed by the different samples. It is obvious that the different feature points have different distributions, while the same feature points in the different samples have similar distributions. The similar feature points are selected to be clustered into one group using the cluster method [9] and to set up the cluster set of the feature points.

### 3.2. Feature Point Cluster

There are 39 feature points in a standard face model *a* and their relative angle histograms of the feature points are denoted by *h*_*i*_(*a*), *i* = 1,2,…, 39. The unlocated face sample is denoted by *b*. The number of the vertex is *L* and the relative angle histogram of each feature point is *h*_*j*_(*b*), *j* = 1,2,…, *L*. Let *N*_*i*_ denote the cluster number of the *i*th feature point, and the steps to determine the *i*th feature point cluster set of the model *b* are as follows.

**Step 1 .**

According to the prior knowledge, the representative points are selected as the initial cluster center CC_*i*_ of each group.

**Step 2 .**

Calculate the similarity between CC_*i*_ and *L* points in the model *b* and sort them in decreasing order.

**Step 3 .**

Select the maximum similarity *N*_*i*_ points as the cluster set *p*_*c*_ of the *i*th feature point in the model *b*.

**Figure 4:**
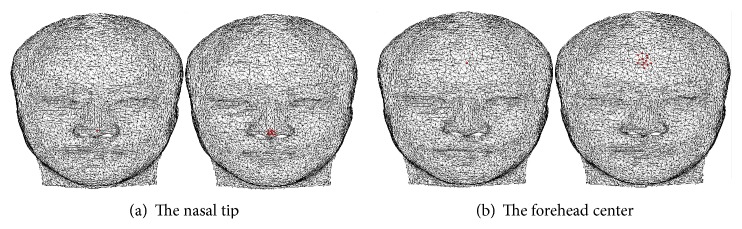
The result of the point cluster.

Figure 4 shows the cluster point set results of the nasal tip and the forehead center in a model whose feature points are to be located. Experiments prove that the fewer cluster points would contain the optimum feature points in the conspicuous feature point place of a face, such as the nasal tip and the mouth corner, while, in the smoother place, such as the forehead center, more cluster points would be needed. The next task is to select precise feature points from the cluster point set.

## 4. Multiscale Integral Features Extraction

### 4.1. Volume Integral Features

The volume integral features are defined as multiscale feature values, which could weaken the noise influence with bigger robustness than the differential values, and measure the concave-convex level of the model surface.

**Figure 5:**
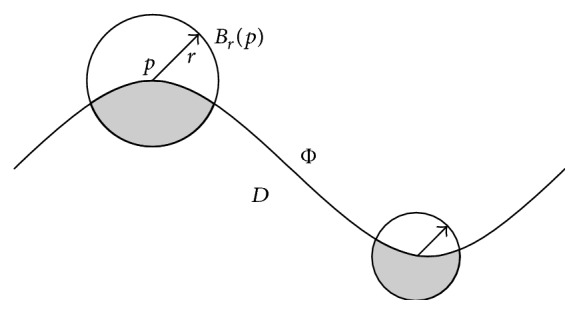
2D representation of the volume integral invariant.

The volume integral invariant of a surface point *p* is defined as a functional integration in the local sphere region of this point [10–12], whose math expression is

(2) $$\begin{array}{c} V^{r}\left(\;p\;\right)=\overset{}{\underset{B_{r}\left(p\right)}{\int }}\chi_{D}\left(\;x\;\right)dx, \end{array}$$

where *B*_*r*_(*p*) is a sphere, which takes *p* as the sphere center and *r* as the radius. As is shown in Figure 5. *χ*_*D*_(*x*) is a surface indicator function. When the point *x* is in the *D* region, *χ*_*D*_ = 1; while *x* is out of the *D* region, *χ*_*D*_ = 0. The geometrical significance of *V* ^*r*^(*p*) is a part of the sphere volume which locates in the lateral of the surface.

Equation (2) is expanded to a polynomial and its limitation value is computed under the condition *r* → 0, so the functional relationship between the volume integral invariant and mean curvature *k*_*H*_ is

(3) $$\begin{array}{c} V_{r}\left(\;p\;\right)=\frac{2\pi }{3}r^{3}-\frac{\pi k_{H}}{4}r^{4}+o\left(\;r^{5}\;\right). \end{array}$$

From (3) we know that the value of the volume invariant is related to the radium of integral sphere region, so the longer the radius is, the bigger the value of volume invariant becomes. Whether there was noise in the interior of the integral region, the impact on the result of the volume invariant is small, so the volume invariant is taken as a feature which possesses robustness. In practice, the geometric feature of the surface on the point *p* is defined as char *V*_*r*_(*p*), which is the ratio of the volume invariant to the volume of sphere region. The equation is

(4) $$\begin{array}{c} char\;V_{r}\left(\;p\;\right)=\frac{V_{r}\left(p\right)}{B_{r}\left(p\right)}. \end{array}$$

According to the calculated values of char *V*_*r*_(*p*), we describe the geometric shape of the surface on the point *p* as convex, approximate plane, and concave. When the scale is *r* and char *V*_*r*_(*p*) < 1/2, the shape of the surface on the point *p* is convex; when char *V*_*r*_(*p*) ≈ 1/2, the shape of the surface on the point *p* is an approximate plane; when char *V*_*r*_(*p*) > 1/2, the shape of the surface on the point *p* is concave.

### 4.2. Multiscale Feature Extraction

**Figure 6:**
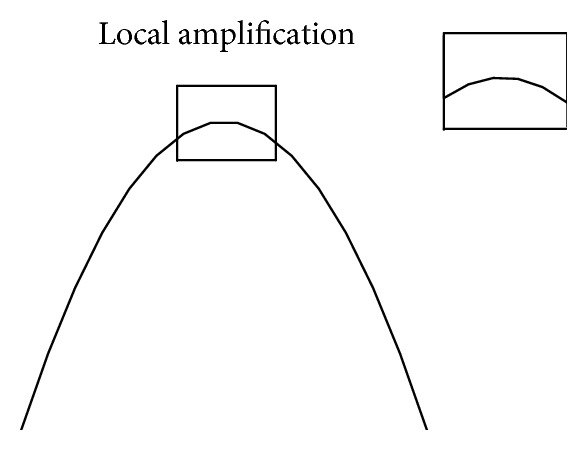
Multiscale theory.

Concave-convex is a relative concept. Concave-convex level will transform with the scale change. As is shown in Figure 6, under a large scale the curve in a box is convex, while under a small scale it will be smooth when the local part is amplified.

**Figure 7:**
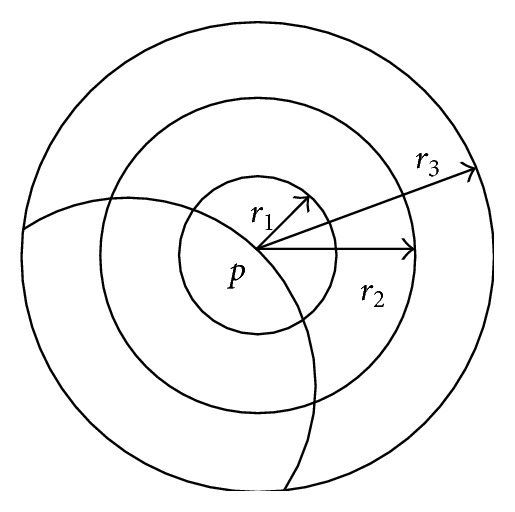
Multiscale feature extraction.

Multiscale geometrical feature extraction [13] means that the feature extraction algorithm extracts the same feature adopting multiple threshold values. As is shown in Figure 7, the surface geometrical features of the point *p* on the surface are extracted under 3 scales: *r*_1_, *r*_2_, and *r*_3_. The large scale can reduce the noise impact more, while the small scale can more precisely evaluate the integral invariant. The large scale should be combined with the small scale in the feature extraction process. Therefore, adopting the multiscale feature extraction method can not only precisely describe the local features but also reduce the noise influence.

### 4.3. Precise Localization of Feature Points

We calculate the integral features of cluster points *p*_*c*_ and point *p* for three different scales of 1, 2, and 3 times of the average side length in the triangular mesh. The distance square root *S* is defined on the integral features. As is shown in (5), every point in *p*_*c*_ is calculated, as well as the *S* of the standard feature points under the three scales. The point *p*_*c*_, the minimum value of *S*, is chosen as a feature point of the model *p*. Consider

(5) $$\begin{array}{c} S=\sqrt{\overset{i=3}{\underset{i=1}{\sum }}{\left(char\;V_{r_{i}}\left(p\right)-char\;V_{r_{i}}\left(p_{c}\right)\right)}^{2}}. \end{array}$$

## 5. Experimental Comparison and Analysis

We design a feature point of 3D face localization system (FaceLS) according to the proposed feature point localization algorithm. Forty sets of 3D point distribution face model data are selected randomly from a monolayer face sample library, which belongs to the North West University, and are taken as the face samples of the feature points which will be located. The algorithm is tested and compared with the localization method of Feng et al. [3]. All the programs are operated by Windows 7, Core i5 processors, 2.8 GHz, 2 G RAM, and Matlab 2010.

The evaluation method of the feature point localization is as follows: given a distance threshold value *ε*, (*X*, *Y*, *Z*) is the accurate position of each feature point in the untested models, and the Euclidean distance *d* between two points can be computed as follows:

(6) $$\begin{array}{c} d=\sqrt{{\left(x-X\right)}^{2}+{\left(y-Y\right)}^{2}+{\left(z-Z\right)}^{2}}. \end{array}$$

The steps of our method are as follows.

**Step 1 .**

According to the prior knowledge, the representative points are selected as the initial cluster center CC_*i*_ of each group.

**Step 2 .**

Calculate the similarity between CC_*i*_ and *L* points in model *b* and sort them in decreasing order.

**Step 3 .**

Select maximum similarity *N*_*i*_ points as cluster set *p*_*c*_ of the *i*th feature point in model *b*.

**Step 4 .**

We calculate the integral features of cluster points *p*_*c*_ and point *p* for three different scales of 1, 2, and 3 times of the average side length in the triangular mesh. The distance square root *S* is defined on the integral features. As is shown in (5), every point in *p*_*c*_ is calculated, as well as *S* of the standard feature points under the three scales. The point *p*_*c*_, the minimum value of *S*, is chosen as a feature point of the model *p*.

**Step 5 .**

Given a distance threshold value *ε*, judge whether the point is correct with (6). If *d* ≤ *ε*, the feature point localization is considered to be correct; otherwise it has a certain error.

**Figure 8:**
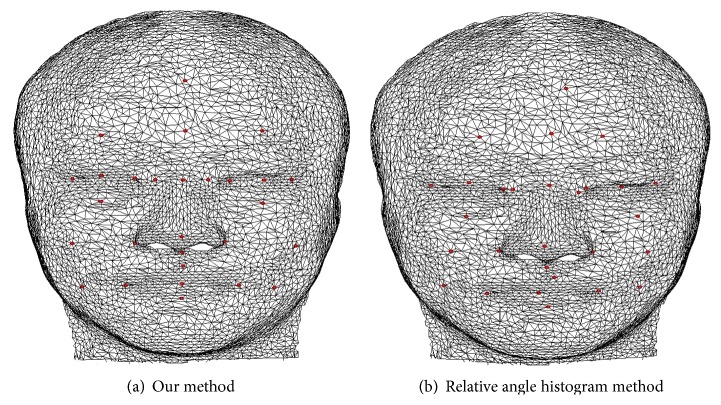
Result comparison of two feature point localization methods.

**Figure 9:**
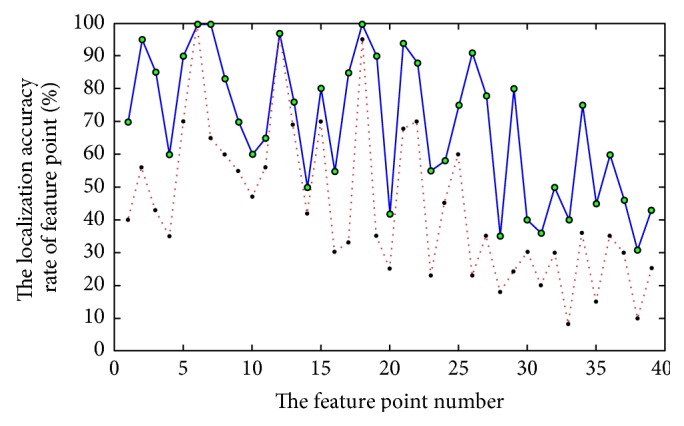
Comparison of the accuracy rate for two feature point localization methods.

Figure 8 shows the results of two methods: one is based on our method and the other is the relative angle histogram localization method of Feng et al. [3] for the same feature point pending model (only 28 feature points are showed, and part of the feature points are coincident from the front view). Using the two localization methods, Figure 9 shows the accuracy rate of 39 feature points in a range of the given distance threshold value. The red dotted line indicates the relative angle histogram localization method of Feng et al. [3], and the blue solid line presents our method. The localization result of our method is more precise.  Table 1 shows the mean accuracy rates of our method are higher than those of Feng et al. [3].

The feature point cluster set is determined firstly by the cluster algorithm and the relative angle histogram algorithm, and then the feature points are located accurately by the steady multiscale integral features, so the accuracy rate of localization is improved greatly. The localization method based on relative angle histograms has limitations, because it locates feature points to a smaller range through comparing the similarity of relative angle histograms and this method is suitable for conspicuous feature points, while the accuracy will be decreased for the inconspicuous feature points.

## 6. Conclusions

In this work we propose a method that combines the relative angle histograms with the multiscale constraints for localization of the feature points in a 3D face. The feature point cluster set is determined through the cluster algorithm and the relative angle histograms, and then feature points are located accurately by the multiscale integral features, which could avoid many wrong matches in the result caused by the precision and similarity of the local geometrical characteristic. Experimental results show that this method performs well and the accuracy rate of localization is improved. But the accuracy rate of localization is nonideal under the condition of feature points on smooth part of a face, so this should be the next research direction.

## Figures and Tables

**Table 1:** The mean accuracy rates of two feature point localization methods.

Method	The mean accuracy rate/%			
	Nose	Left eye	Right eye	Mouth
Feng et al. [3]	62.0	78.3	77.6	63.8
Our method	89.2	84.5	85.6	71.3
